# STID-Net: Optimizing Intrusion Detection in IoT with Gradient Descent

**DOI:** 10.3390/s25061852

**Published:** 2025-03-17

**Authors:** James Deva Koresh Hezekiah, Usha Nandini Duraisamy, Kalaichelvi Nallusamy, Avudaiammal Ramalingam, Saranya Chandran, Murugesan Rajeswari Thiyagupriyadharsan, Periasamy Selvaraju, Rajagopal Maheswar

**Affiliations:** 1 Department of Electronics and Communication Engineering, Centre for IoT and AI (CITI), KPR Institute of Engineering and Technology, Coimbatore 641 407, Tamil Nadu, India; jamesdevakoresh@gmail.com; 2 Department of Computer Science and Engineering, Sathyabama Institute of Science and Technology, Chennai 600 119, Tamil Nadu, India; usha.cse@sathyabama.ac.in; 3 Department of Information Technology, Sri Sivasubramaniya Nadar College of Engineering, Kalavakkam 603 110, Tamil Nadu, India; kalaichelvin@ssn.edu.in; 4 Department of Electronics and Communication Engineering, St. Joseph’s College of Engineering, Chennai 600 119, Tamil Nadu, India; avudaiammalr@stjosephs.ac.in; 5 Department of Electronics and Communication Engineering, Ashoka Women’s Engineering College, Kurnool 518 002, Andhra Pradesh, India; naveensaranyac@gmail.com; 6 School of Electrical and Electronics Engineering, VIT Bhopal University, Sehore 466 001, Madhya Pradesh, India; thiyagu.priyadharsan@gmail.com; 7 Department of Computer Science and Engineering, Saveetha School of Engineering, Saveetha Institute of Medical and Technological Sciences (SIMATS), Thandalam, Chennai 602 105, Tamil Nadu, India

**Keywords:** cybersecurity, anomaly detection, feature optimization, pattern recognition, sequential data analysis

## Abstract

The rapid evolution of IoT environment in medical and industrial applications has led to an increase in network vulnerabilities, making an intrusion detection system a critical requirement. Existing methods often struggle in capturing complex and irregular patterns from dynamic intrusion data, making them not suitable for different IoT applications. To address these limitations, this work proposes STID-Net that integrated customized convolutional kernels for spatial feature extraction and LSTM layers for temporal sequence modelling. Unlike traditional models, STID-Net has an improved ability to identify irregular patterns in dynamic datasets. This work is also equipped with an attention mechanism for enhancing the detection of long-term dependencies in intrusion patterns. The STID-Net is also experimented with the MBGD and SGD optimizers, and we are satisfied with the performance of the SGD optimizer in both the IoMT and IIoT datasets. The SGD optimized model provides a faster convergence and better weight adjustments for handling noisy datasets, making it robust and scalable for diverse IoT applications. This experimental work demonstrates an accuracy of 97.14% and 97.85% with the MBGD optimizer in the IoMT and IIoT datasets, while it attained 98.58% and 99.15% with SGD optimization, respectively. The proposed methodology also outperforms the standalone CNN and LSTM models incorporated with both optimizers, and the result indicates the robustness and scalability of STID-Net in medical and industrial applications.

## 1. Introduction

In cybersecurity, intrusion detection systems (IDSs) are employed to detect and respond to malicious activities and unauthorized access to a network or host system. Basically, intrusion detection systems are categorized into two types: network-based IDSs (NIDSs) and host-based IDSs (HIDSs). NIDSs are structured to be placed at different locations of a network for analyzing the network traffic in real time from the monitored data. It achieved this by inspecting the packets transmitted on the network based on its performance on unauthorized access, port scans, denial of service (DoS) and other network-related attacks. This inspection is carried out over the packets before they enter into their respective host elements. Thus, NIDSs are acting as a protective and defensive layer for any network infrastructure. On the other side, HIDSs are utilized for individual host machines to monitor and analyze the login, system call and file integrity on the connected hardware or network system. Hence, HIDSs have the ability to detect system level anomalies and unauthorized actions. HIDSs are also used for providing information on the behaviour of the system on malware, insider threats and other host-specific attacks. However, NIDSs are widely preferred due to their effectiveness on providing both network and hardware level intrusion protection. Networks incorporated with NIDSs and HIDSs enhance the security capability of a network in different layers of operation.

Before IDSs, several firewalls, blockchain-based smart contracts [1] and antivirus software were used in network systems for classifying the cyberthreats. In general, ensemble methods perform well for any classification and forecasting kind of applications [2,3]. Though there are some existing software solutions, they are not sufficient for providing protection against sophisticated attacks. IDSs are found to be satisfied as it identifies both known and unknown threats by using pattern- and signature-based detection techniques. The involvement of trending methodologies like machine learning and artificial intelligence with IDSs makes the network to be efficient against complex and evolving attack vectors.

### 1.1. Significance of IoMT and IIoT in Modern Applications

In recent days, the advancements of IoMT represents a remarkable growth in healthcare industries, where it provides enhanced patient care, improved operational efficiency and reduced healthcare costs. It is achieved by integrating several medical devices and systems for real-time data collection and transmission. IoMT encompasses a continuous analyzing system for identifying the vital signs of patients even for remote applications. Hence, it opens a door for personalized treatment plans that improve the reliability of smart implants, wearables and remote patient monitoring systems. IoMT are found to be successful for chronic disease management and the incorporation of healthcare system; IoMT facilitates preventive care for the patients and reduces hospital readmissions along with a better patient outcome. Moreover, the efficiency of IoMT is improved to a certain level by connecting them with various medical devices for automated and streamlined workflows to ensure better resource management. Automated medication dispensers and smart infusion pumps are examples of medical devices that are majorly connected with IoMT systems for ensuring accurate dosage delivery with minimal human error. Additionally, IoMT enables an opportunity for sharing the collected healthcare data with different forecasting systems to provide reliable decisions on treatment. This interconnection supports advanced data analytics for providing valuable insights towards overall patient care and disease outbreaks.

The Industrial Internet of Things (IIoT) transforms the traditional industrial setup through the integration of smart technologies and seamless connectivity to ensure reliable operation, productivity and safety. IIoT allows real-time data collection from industrial machines, sensors and control systems for monitoring and analytics via the help of internet connectivity. This facility makes the system perform predictive maintenance by monitoring unusual signs from the wear and tear of the machinery. It helps the operator to prepare a scheduled maintenance that reduces the downtime of the system. The lifespan of the equipment is also improved through IIoT by preventing unexpected breakdowns and improving production process optimization.

Furthermore, IIoT facilitates an improved resource management system for energy and water usage in industries by implementing smart sensors on the connected systems to ensure optimal utilization and waste reduction. IIoT enhances the precision of a complex operation through automation by providing consistency in its production. The real-time analytics of IIoT allows the system to do immediate adjustments on the production parameters, ensuring high quality outcomes. Moreover, IIoT improves supply chain management by having real-time visibility on the inventory, logistics and delivery schedules, thereby ensuring a better coordination towards the market demands. IIoT provides a significant impact on industrial safety by monitoring environmental hazards, thereby preventing accidents. It is achieved by making automated responses and triggers that detect the potential risks. Industries like oil and gas, mining and chemicals enforce IIoT models to ensure the safety of their workers by monitoring the conditions of systems based on compliance and safety protocols. The ability of IIoT systems to analyze vast data in real time pushes the industrial sector toward innovations, initiatives and improvements. Overall, IIoT revolutionizes the industrial sector through its smarter solution on operation and safety, leading to profitability and sustainability.

### 1.2. Role of IDSs in IoMT and IIoT

IDSs have become essential for IoMT for ensuring the integrity and security of interconnected medical devices to the networks. IoMT devices are designed to send sensitive data continuously from a patient or medical device to a remote monitoring system or station for storage and further analysis. In general, IDSs of IoMT are designed to monitor the anomalies and unauthorized activities on a device’s communication layer and the network layer. Continuous data transmission might make the network as busy as possible and make the firewalls insufficient for the process of intrusion detection. Due to the nature of handling high-density data, an IDS performs well in such scenarios by analyzing the patterns on the transmission data through big data analysis. The algorithm of IDS is also structured for generating timely alerts and a response algorithm for preventing the leakage of data to cyberattacks. The implementation of an IDS in IoMT is critical as it needs to maintain the most sensible medical data with confidentiality and compliance towards connected healthcare systems.

In IIoT, IDSs are utilized to avoid cyber threats on industrial systems and software. IIoT integrates internet connectivity with sensors, control systems and industrial machineries that allow for intelligent automation and real-time data analytics. Though this internet-based process improves operational efficiency, it also creates a way to open cyber vulnerabilities to the system. IDS are trained to identify potential threats by analyzing network traffic, device-to-device communication and system activities. Signature-based detection, behavioural analysis and anomaly detection are the most utilized approaches in IDSs for detecting unauthorized access and disruption over the IIoT. The early warning received from an IDS makes the system sustainable by protecting critical industrial processes. IDSs are required for safeguarding intellectual property and advancing smart manufacturing processes. Additionally, it improves the energy efficiency and reliability of industrial operations. Though the existing techniques were developed with good accuracy on intrusion detection, they lag the efficacy needed for detecting the long-term temporal dependencies and irregular patterns in IoT data. Hence, it leads to a reduction in accuracy attainment in certain dynamic intrusion scenarios.

Techniques like gradient descent have shown potential for faster training and resource optimization, but they require further enhancements to meet the demands of modern IoT ecosystems. This highlights the need for a universal deep learning framework that balances accuracy across diverse IoT applications. This research introduces STID-Net, a novel architecture that combines spatial and temporal learning with an attention mechanism to address the limitations of traditional deep learning models in intrusion detection in dynamic intrusion scenarios like medical and industrial IoT applications. The following section represents the attainments of the recent year machine learning and deep learning methodologies that were developed for intrusion detection in medical and industrial IoT scenarios.

## 2. Related Work

### 2.1. Review on Machine Leaning Methods

A Meta-IDS was developed to identify the known and zero-day threats through signature- and anomaly based detection strategies. The methodology is also incorporated with a privacy-preserving technique for handling the precious medical information available in the WUSTL EHMS 2020 dataset. The experimental work indicates an accuracy of 99.57% and 99.47% with the signature-based detection and anomaly based detection methods, respectively [4]. An extreme gradient-boosting technique was proposed to identify intrusions in the WUSTL EHMS 2020 dataset, and its experimental study indicates an accuracy of 95.01% when selecting the data features through a maximum information coefficient approach. The maximum information coefficient approach is employed in this work to analyze the nonlinear relationships available in the dataset [5]. An ensemble model called FusionNet was designed to achieve a 98.5% accuracy on the WUSTL EHMS 2020 dataset. The FusionNet model leverages the SVM, KNN, random forest and multi-layer preceptor algorithms for its architecture [6]. A multi-phase architecture was structured using federated learning with an XGBoost meta-learning model for identifying intrusion in the WUSTL EHMS 2020 dataset. The experimental analysis represents an accuracy of 99.45% [7]. An XGBoost classifier was optimized with a genetic algorithm for finetuning its hyperparameters for intrusion detection from the WUSTL EHMS 2020 dataset. The dataset features are encoded by an autoencoder in this work for attaining an accuracy rate of 98.98% [8].

The random forest classifier was used to detect anomalies in the WUSTL IIoT 2021 dataset. The work utilizes Pearson’s coefficient and isolation forest for minimizing the computational cost of the proposed work. The isolation forest technique removes the outliers from the dataset, and Pearson’s coefficient is implemented for selecting the best features for the training process. The experimental report states an accuracy of 99.12% and 93.96% with the Matthews correlation coefficient [9]. The random forest classifier was utilized to detect malicious behaviours on the WUSTL IIOT 2021 dataset. The performance of the classifier was validated with two different optimizers, namely the bat algorithm and the particle swarm optimization. The experimental work observes an accuracy of 99.99% with the random forest classifier equipped with the bat algorithm and 95.68% with the particle swarm optimization algorithm [10]. A machine learning technique using gaussian naive bayes and stochastic gradient descent was developed to detect intrusions in the WUSTL EHMS 2020 dataset. Principal component analysis along with a singular value decomposition is included in this work for minimizing the computational burden of the model. The suggested model attains an accuracy of 96% [11]. The performance of the stochastic gradient descent classifier was verified with four feature selection algorithms developed based on a ride regressor. This model achieved an accuracy of 92.69% when the data features are reduced to 79.93% [12]. An ensemble technique was constructed using lightweight machine learning models like Gaussian naïve bayes, decision tree and logistic regression as the base classifier, and they included the stochastic gradient descent as the meta classifier for detecting network intrusions in public datasets. The experimental work incorporates the Chi-square test for identifying the most relevant features from the dataset for its classification process. The experimental work indicates an accuracy of 99.84%, 93.88% and 99.8% for the KDD 99, UNSW-NB15 and CIC-IDS2017 datasets [13].

### 2.2. Review on Deep Leaning Methods

An optimized convolutional neural network approach was designed to detect intrusions in the IIoT environment. Here, a systematic optimization technique is used for improving the performance of the CNN during its training process with a balanced dataset. The WUSTL IIOT 2021 dataset was utilized in this work with the SMOTE methodology for ensuring an equal representation of data in all classes. The result demonstrates an accuracy of 99.9% [14]. A hybrid deep learning approach combining convolutional neural network and gated recurrent unit along with a Bayesian optimization technique for fine tuning its hyperparameters was proposed. This work achieved an accuracy rate of 97.68% along with a precision of 97.67% with the WUSTL IIOT 2021 dataset and 97.8% with the Edge IIoT dataset [15]. A deep reinforcement technique was suggested to identify anomalies in the SCADA network. The work utilizes the Q-network for learning complex patterns from the dataset, and the model was trained with 25 networking features and attained an accuracy of 99.36% with the WUSTL IIoT 2021 dataset [16]. A deep neural network-based classification technique was proposed to identify malicious network activity. The work utilizes the Jacobian saliency map for identifying the best features from the dataset, and the work includes stochastic gradient descent and the Adam optimizer for the optimization of parameters in the classifier. The experimental work shows a better performance of 98.97 for the F1 score in the DDoS 2019 dataset [17]. The long short-term memory approach was utilized to detect wireless intrusions, and the extreme gradient boosting algorithm was incorporated to identify the optimum features from the dataset to improve its outcome. The methodology identified 17 best features from the UNSW-NB15 dataset and 22 best features from the NSL-KDD dataset. The experimental work attains a best accuracy of 99.49% in the NSL-KDD dataset [18]. An RMD-Graph model was proposed to enhance malicious domain detection through a dual denoising module incorporated with an autoencoder. Through graph contrastive learning, the model provides a self-supervised learning environment with a highly adaptable module. Experimental results indicate a betterment on a public DNS dataset over the baseline methods [19]. The KNN model was incorporated with a CNN and the gorilla troops optimizer for feature extraction and selection to construct a hybrid intrusion detection system. The performance of the work was verified with the NSL-KDD dataset, and it found an accuracy of 99.86% [20].

### 2.3. Problem Statement

Here, this review indicates that most of the previous methodologies used machine learning algorithms, and they attained a decent accuracy on intrusion detection in the WUSTL EHMS 2020, WUSTL IIOT 2021 and other datasets. However, there was no information about how the available computational resources were utilized in the previous works. In general, an efficient optimization of computational resources plays a crucial factor on the real-time implementation of a deep learning algorithm. Additionally, the efficient resource allocation method enables the intrusion detection system to be adopted with more complex neural network algorithms despite a limited memory and computational capability. Techniques like gradient descents [11,12,17] are widely preferred for such optimizations, allowing the neural network models to be trained faster with a decent accuracy. This is highly important for implementing intrusion detection systems in medical and industrial applications. The proposed work aims to improve the accuracy along with a better resource allocation and detection speed over the existing methods. Additionally, there is a need for a universal deep learning technique that can find IoT intrusions in different applications.

### 2.4. Contributions of the Proposed Work

This research work proposes STID-Net for enhancing intrusion detection in the IoMT and IIoT networks. Existing research in intrusion detection primarily focuses on achieving higher accuracies, and they often neglect to capture dynamic and long-term dependencies in the IoT data. The proposed architecture addresses the research limitations of the traditional deep learning models by integrating both spatial and temporal learning with an attention mechanism for optimizing the feature extraction process in an efficient manner. The work has a customized convolutional kernel with SGD for achieving superior performance in handling complex and irregular patters from the time series IoT data. It further optimizes computational resources through customized kernels and optimization techniques to ensure a better performance in a real-time resource-constrained IoT environment. The key highlights of this work is as follows:

- STID-Net combines CNN for spatial feature extraction and LSTM for temporal sequence modelling, offering a comprehensive solution for detecting anomalies in highly dynamic intrusion data;
- The proposed work introduces customized kernel sizes for optimizing feature irregularities and incorporates an attention mechanism for assigning higher relevance to critical temporal patterns to improve the detection of long-term dependencies in the IoT data;
- The performance of the architecture is verified with both MBGD and SGD, enabling a precise weight adjustment for enhancing the robustness and scalability in handling noisy data from the IoTM and IIoT environments.

These contributions collectively establish STID-Net as a state-of-the-art solution for addressing the evolving security challenges in IoT ecosystems.

## 3. Methodology

**Figure 1:**
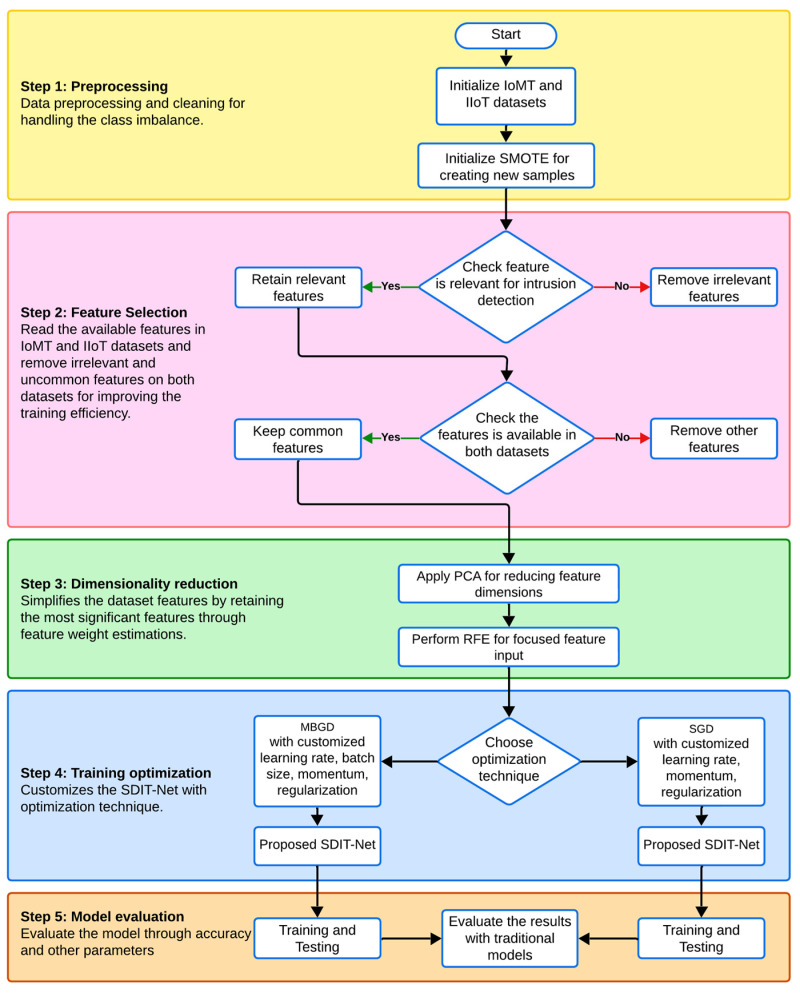
Workflow of the proposed work.

The workflow of the proposed study can be classified into four sections: data preprocessing, feature selection, dimensionality reduction, training optimization and model selection or the testing of deep learning classifiers. Figure 1 represents the graphical representation and the workflow of the proposed work, and each block is explained in the following sections.

### 3.1. Data Preprocessing and Cleaning

Preprocessing is the first step included in the workflow for ensuring the consistency of the dataset by addressing the missing values through a data normalization method. In the proposed work, the missing values are replaced by estimating a mean value from their respective features, ensuring an unaffected data distribution. Additionally, data normalization ensures that the missing scale features are performed within a specific range, thereby reducing the risk for a biassed outcome caused by data variations in feature magnitudes. Moreover, redundant features are removed from the dataset, reducing the training complexity. The WUSTL EHMS 2020 [21] and WUSTL IIOT 2021 [22] datasets are used in this work as they provide comprehensive and real-world data from medical and industrial IoT environments. The WUSTL EHMS 2020 dataset includes medical data, allowing the model to assess security from a sensitive healthcare system, whereas the WUSTL IIOT 2021 dataset offers data from industrial IoT applications. This enables testing of the proposed work in different environments with varying levels of complexity and resource constraints. These datasets are also crucial for ensuring the dynamic applicability of the proposed STID-Net in both medical and industrial domains.

Since this study is designed to classifying the intrusions with binary classifications, least count intrusions like Backdoor and CommInj available in the WUSTL 2021 IIoT dataset are completely removed. However, the SMOTE (synthetic minority over-sampling) technique is applied to the remaining all-attack samples for efficient data normalization. SMOTE has the ability to generate new samples for the minority class attacks equal to the count of the normal class attacks available in both the IIoT and IoMT datasets.

Since the normal class samples are always higher in the intrusion datasets, it is essential to have a SMOTE kind of sampling technique to balance such an imbalanced dataset. Therefore, SMOTE allows the deep learning networks to learn better decision boundaries on each class through the augmentation of the minority class samples. Hence, the risk of observing biassed output is rectified in the deep learning classifications. Mathematically, the process of generating new samples through SMOTE can be represented as follows:

(1) $$x_{new\_sample}=x_{a}+R\;(x_{a,b}-x_{a})\;$$

where

$x_{a}$ = random minority sample,

$x_{a,b}$ = k nearest neighbour of the minority sample and

R = random number range.

Additionally, a data cleaning process is also included in this work; it keeps only the features that are commonly available in both the IIoT and IoMT datasets. This enables the design of a deep learning technique with a customized optimization technique for detecting intrusions in both the datasets effectively. The list of features that are finally considered in this work is listed in Table 1. These features are commonly available in both datasets.

### 3.2. Feature Extraction

Principal component analysis (PCA) is a powerful dimensionality reduction technique employed in this work for enhancing the classification process by simplifying the dataset while retaining the most significant variance available in it. In particular, PCA is required in the proposed work for mitigating the effects of noisy features and multicollinearity in the IoMT and IIoT datasets. It also reduces the dimensionality of the dataset to a lower dimensional space by retaining the most significant features. The process begins by centering the data through a subtraction of the mean vector, resulting in a centred data matrix. Next, the covariance matrix of the data is computed, and eigenvalue decomposition is performed to identify the principal components. These components represent the orthogonal directions of the maximum variance in the dataset feature. The top components with the largest eigenvalues are selected and considered as the significant feature. This process helps in eliminating redundant and noisy features, preserving only the most relevant information for intrusion detection. This also reduces the risk of overfitting and computational overhead.

Primarily, PCA transforms the original feature space of the dataset into a new set of orthogonal axes called principal components, which are the linear combinations extracted from the original features extracted from raw data. The process begins with cantering the data through the subtraction of the mean vector from the original dataset, resulting in a cantered data matrix as follows:

(2) $$X^{\prime }=X-\mu$$

where

X = original data matrix,

μ = mean vector of the dataset and

$X^{\prime }$ = centred data matrix.

Next, the covariance matrix is estimated to make an assessment on the feature variance process. Additionally, an eigenvalue decomposition is included for estimating the amount of variance estimated by each principal component as follows:

(3) $$C=\frac{1}{n-1}{\left(X^{\prime }\right)}^{T}X^{\prime }$$

(4) Cv=λv

where

C = covariance matrix,

n = number of samples,

λ = eigenvalues and

v = eigenvectors.

This indicates that the highest eigenvalues are associated with the most significant variance directions in the data. The transformation of the original dataset into a new feature space is accomplished by projecting it on the highest eigenvectors, resulting in a reduced feature matrix. Therefore, the new feature space can be represented as

(5) $$Z=X^{\prime }V_{k}$$

where

Z = matrix of reduced features and

$V_{k}$ = matrix containing the top eigenvectors.

Hence, the dimensionality of the dataset is reduced significantly, thereby improving the training process by preserving the essential patterns of the available dataset. This is particularly required in this work, where the high dimensional input of the dataset can improve the possibility of overfitting, which leads to an improved computational complexity. Ultimately, PCA improves the robustness of the proposed work to provide a better detection on the anomalies.

### 3.3. Feature Selection

Recursive feature elimination (RFE) is employed along with PCA for improving the feature selection process to provide an efficient and focused input to the deep learning architectures. Additionally, PCA performs feature extraction by transforming the information available in the dataset into an orthogonal component to retain the most important features, whereas RFE is a feature selection method employed for identifying and utilizing only the most relevant features required for classification in a particular application. The combination of PCA and RFE provides a streamlined feature set for the deep learning algorithm for its training process.

RFE reduces the feature dimensionality by calculating the feature weights with respect to the coefficient of the model or its application. At each iteration, RFE eliminates the least significant feature with respect to the score calculated from the model’s coefficient. Furthermore, RFE repeats this iteration process until it identifies the desired number of features. Mathematically, a classifier with parameters ω and features $X=\left\{x_{1},x_{2},x_{3}\ldots ,x_{n}\right\}$ estimates the importance of each feature by examining the weight $\omega_{i}$ with respect to the feature associated with $x_{i}$. Therefore, the feature elimination criterion can be defined as

(6) $$x_{least}=arg\min\left|\omega_{i}\right|$$

where $x_{least}$ is the feature with the smallest weight or importance score which is removed during the iteration.

Hence, RFE identifies a selected set of features, $X_{selected}$, that contain only the most impactful features for the required application by reducing the noisy and redundant features from the dataset. Moreover, the combination of PCA and RFE enables the classifier model to perform in a faster and efficient way as they are trained with highly informative features.

### 3.4. Deep Learning Models Overview

In this work, the performance of convolutional neural networks (CNNs), long short-term memory networks (LSTMs) and the proposed CNN-LSTM are effectively analyzed for identifying a better neural network algorithm for intrusion detection in the IoMT and IIoT datasets. These models were particularly selected for their ability to capture spatial and temporal patterns in detecting anomalies from a complex data structure. Overall, these models are selected for their effectiveness in input data comparison with the reconstruction data. A detailed technical overview of each model including their architectural and mathematical principles is discussed in the following sections.

#### 3.4.1. Convolutional Neural Networks

The CNN model is employed in this work for its effectiveness on extracting spatial features from structured datasets like IoMT and IIoT that contain intrinsic spatial hierarchies. Therefore, CNN has the ability to identify localized patterns, which are primarily required for detecting anomalies and cyber-attacks, within the network traffic. Generally, CNN is structured with multiple convolutional layers followed by an activation function and pooling layers. Hence, the convolutional layer acts like a filter for the input data to generate feature maps from the captured spatial dependencies.

**Figure 2:**
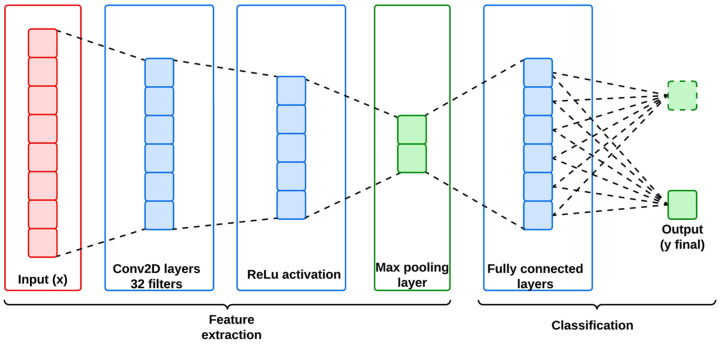
Architecture of CNN.

The convolutional operation of the CNN can be expressed mathematically as follows:

(7) $${Feature\;Map}_{i,j}=\sum_{m=0}^{m-1}\sum_{n=0}^{n-1}{Input}_{i+m,j+n}\cdot {Filter}_{m,n}$$

where M and N are representing the dimensions of the filter, and i,j are the spatial indices. Here, the feature maps are down-sampled using pooling layers to reduce the dimensionality and computational complexity of the identified feature data from the dataset. Mathematically, the pooling operation can be represented as follows:

$$y\left[i,j,k\right]=\underset{m,\mathrm{n\epsilon window}}{\max}x[i+m,j+n,k]$$

where *k* indicates the filter bias. Furthermore, the identified feature data are subsequently passed through a fully connected layer for classification from the learned spatial hierarchies. Figure 2 represents the architecture of the utilized CNN model, and its mathematical workflow is descripted below in Algorithm 1.
**Algorithm 1:** Mathematical workflow of the utilized CNN**Step 1:** Input layer (x)$\mathrm{Input}\;x\;\in \;R^{N,\;\;H,\;W,\;C}$ Here, N is the batch size; H and W are the spatial dimensions, and C is the number of channels.**Step 2:** Convolutional layer   Apply convolution operation to extract features:$F_{CNN}=ReLU\;\left(\sum_{C=1}^{C}W_{c}*X_{c}+b_{c}\right)$$\;\;\;\mathrm{Here},\;W_{c}$$\;\mathrm{is}\;\mathrm{the}\;\mathrm{convolutional}\;\mathrm{kernel}\;\mathrm{for}\;\mathrm{channel}\;c,\;and\;b_{c}$ is the bias term.$output=F_{CNN}\in R^{N,H^{\prime },\;W^{\prime },\;K}$   Here, K$\;\mathrm{is}\;\mathrm{the}\;\mathrm{number}\;\mathrm{of}\;\mathrm{filters},\;\mathrm{and}\;H^{\prime }\;and\;\;W^{\prime }$ are the reduced spatial dimensions.**Step 3:** Activation ReLU$ReLU\;\left(x\right)=\max\left(0,\;x\right)$**Step 4:** Pooling layer   Reduce spatial dimensions using max pooling:$F_{Pool}=MaxPool\;(F_{CNN})$$output=F_{CNN}\in R^{N,H^{\prime \prime },\;W^{\prime \prime },\;K}$$\;\;\;\mathrm{Here},\;H^{\prime \prime }\;and\;\;W^{\prime \prime }$ are the further-reduced dimensions.**Step 5:** Fully connected layer   Flatten pooled features and apply a dense layer:$y_{dense}=ReLU\;(W_{d}{\;F}_{pool}+b_{d})$$output=y_{dense}\in R^{N,D}$   Here, D is the number of units in the dense layer. **Step 6:** Output layer   Apply softmax for classification:$y_{final}=Softmax\;(W_{o}y_{dense}+b_{o})$$output=y_{final}\in R^{N,C}$   Here, C is the number of classes.

#### 3.4.2. Long Short-Term Memory Networks

**Figure 3:**
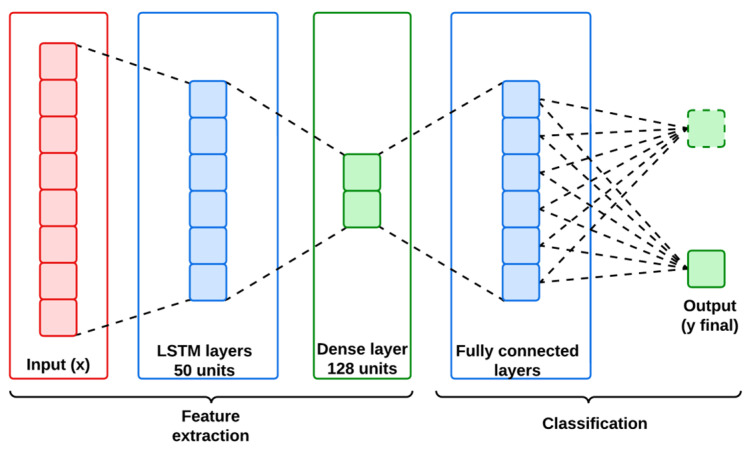
Architecture of LSTM.

LSTMs are the customized version of recurrent neural networks (RNNs) designed to handle sequential and temporal data by maintaining long-term dependencies. This capability is important for handling the IoMT and IIoT datasets, where understanding the temporal dynamics of network traffic is complex but necessary for classifying the abnormal patterns of cyber attacks. An LSTM network is structured with multiple LSTM cells for regulating the flow of information between the input and the output layer. Figure 3 represents the architecture of the LSTM model used in this work.

The key components of LSTM include input gate, forget gate and output gate which controls the cell state $C_{t}$ and hidden state $h_{t}$. Figure 3 represents the architecture of the LSTM model used in this work, and its mathematical workflow is descripted below in Algorithm 2.
**Algorithm 2:** Mathematical workflow of the utilized LSTM**Step 1:** Input layer**Input** $x\;\in \;R^{N,\;\;T,\;F}\;$   Here, N is the batch size; T is the number of steps, and F is the feature dimension. **Step 2:** LSTM cell   For each time step T, compute the following:   Forget gate:$f_{t}=\sigma (W_{f}\cdot \left[h_{t-1},x_{t}\right]+b_{f})$   Input gate:$i_{t}=\sigma \left(W_{i}\cdot \left[h_{t-1},x_{t}\right]+b_{i}\right)$   Candidate memory:${\overset{˘}{C}}_{t}=\sigma \left(W_{C}\cdot \left[h_{t-1},x_{t}\right]+b_{C}\right)$   Update memory state:$C_{t}=f_{t}*C_{t-1}+i_{t}*{\overset{˘}{C}}_{t}$   Output gate:$o_{t}=\sigma \left(W_{o}\cdot \left[h_{t-1},x_{t}\right]+b_{o}\right)$   Hidden state:$h_{t}=o_{t}*tanh(C_{t})$$output:H=[h_{1},\;h_{2},\ldots ,h_{t}]\in \;R^{N,\;\;T,\;F}\;$   Here, U is the number of LSTM units.**Step 3:** Fully connected layer   Flatten features and apply a dense layer:$y_{dense}=ReLU\;(W_{d}F_{attention}+b_{d})$$output:y_{dense}\in R^{N,D}\;$**Step 4:** Output layer   Apply softmax for classification:$y_{final}=Softmax\;(W_{o}y_{dense}+b_{o})$$output=y_{final}\in R^{N,C}$   Here, C is the number of classes.

These equations indicate how LSTM captures data to retain the temporal dependencies, allowing the model to be adopted for modelling the sequential nature of both IoMT and IIoT datasets for anomaly classifications.

#### 3.4.3. Proposed Spatio-Temporal Intrusion Detection Network (STID-Net)

The proposed STID-Net is a hybrid model designed to synergize the strengths of both CNN and LSTM to specifically address the network security issues in IoMT and IIoT. This hybrid model is designed to integrate spatial feature extraction with temporal sequence modelling. Hence, it offers a comprehensive framework for detecting complex patterns and anomalies in time series and sequential data. The proposed work also customizes the kernel size for optimizing the feature irregularities from the irregular patterns on the intrusions. Additionally, an attention layer is also incorporated in this work to improve the ability to capture long-term dependencies by assigning a higher relevance to critical intrusion data.

**Figure 4:**
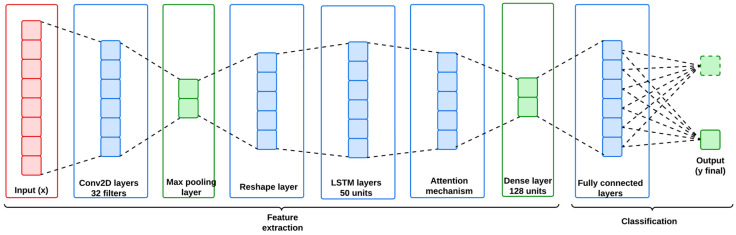
Architecture of the proposed STID-Net.

Figure 4 indicates the architecture of the proposed STID-Net, and the mathematical model and the layer flow of the proposed STID-Net are discussed below in Algorithm 3. It outlines the architecture of STID-Net, highlighting the integration of customized convolutional kernels and an attention mechanism.
**Algorithm 3:** Mathematical workflow of the proposed STID-Net**Step 1:** CNN feature extraction (spatial feature learning)$F_{CNN}=Conv2D\;(X,Filters=32,Kernel\;Size=\left(3,3\right),Activation=ReLU)$**Input: **$X\in R^{\left(N,H,W,C\right)}$   Here, N is the batch size; H×W represents the spatial dimensions, and C is the number of channels.**Output: **$F_{CNN}\in R^{\left(N,W^{\prime },H^{\prime },32\right)}$   The convolutional layer is employed to domain-specific kernels designed to detect anomalies and patterns in the IoMT and IIoT data with a value of 32. **Step 2:** Maximum pooling layer$F_{Pool}=MaxPool2D\;(F_{CNN},Pool\;Size=\left(2,2\right))$$\;\;\;\mathrm{Reduces}\;\mathrm{Spatial}\;\mathrm{Dimensions}:\;F_{Poll}\in R^{\left(N,H^{\prime \prime }/2,W^{\prime \prime }/2,32\right)}$**Step 3:** Reshape layer$F_{reshaped}=Reshape\left(F_{Pool}\right)$**Output: **$F_{reshaped}\in R^{\left(N,T,32\right)}$   Here, T represents the time steps.**Step 4:** LSTM sequence modelling (temporal dependency learning)$h_{t},c_{t}=LSTM\;(F_{reshaped},Units=50)$**Output: **$H\in R^{\left(N,T50\right)}$$\;\;\;\mathrm{Here},\;h_{t}$$\;\mathrm{is}\;\mathrm{the}\;\mathrm{hidden}\;\mathrm{state},\;\mathrm{and}\;c_{t}$ is the cell state.**Step 5:** Attention mechanism integration (novelty)$A_{t}=Softmax\;(W_{a}\cdot H+b_{a})$$F_{attention}=A_{t}\cdot H$**Output 1: **$A_{t}\in R^{\left(N,T\right)}$**Output 2: **$F_{attention}\in R^{\left(N,T,50\right)}$   The attention mechanism assigns dynamic weights to time steps for highlighting the critical patterns. **Step 6:** Dense layer (final decision)$y_{dense}=Dense\left(F_{attention},Units=128,Activation=ReLU\right)$**Output: **$y_{dense}\in R^{\left(N,128\right)}$**Step 7:** Output layer (classification)$y_{final}=Softmax\;(y_{dense},Units=2)$**Output: **$y_{final}\in R^{\left(N,2\right)}$

This final output represents the probability distribution across the classes normal and malicious.

STID-Net starts with the CNN layers, and it extracts the spatial features from the input data through its convolutional filters to identify the local patterns on the anomalies. After convolution, a pooling operation is introduced in this work to reduce the dimensionality of the feature maps by preserving the major features. This step helps the proposed work to address the computational complexity issue in a perfect way. STID-Net is also equipped with a customized convolutional kernel for handling the domain-specific characteristics to extract the spatial feature in an efficient way from the IoMT and IIoT datasets. Furthermore, the output from the CNN layers is reshaped to meet the input requirements of LSTM. LSTM extracts the temporal dependencies and sequential patterns from the reshaped data. It maintains both the hidden state and the cell state from the patterns, enabling the model to learn and retain information over long sequences. The ability to understand both short-term and long-term dependencies is important for identifying the trends and subtleties in the anomalies that are emerging over time.

An attention mechanism is introduced in this work to assign dynamic weights on different time steps in the sequence, thereby enabling the model to focus on the most relevant portions in the dataset. The attention mechanism increases the accuracy and robustness of the proposed work by highlighting the critical moments and patterns in the anomalies. After LSTM and the attention mechanism, the output is forwarded to a fully connected dense layer to map the output space from the extracted features. Finally, the network is concluded with a softmax output layer, providing probabilistic classification for classifying normal and malicious traffic in the network. The combination of spatial and temporal feature learning coupled with dynamic attention makes STID-Net a powerful tool for tackling the dynamic nature of the IoT environments.

### 3.5. Integration of Gradient Descent Techniques with Deep Learning Models

The deep learning models employed for analysis in the proposed work are trained using two different customized optimization methods, namely mini-batch gradient descent (MBGD) and stochastic gradient descent (SGD). Therefore, the convergence speed and accuracy of these deep learning models are fine tuned to classify the anomalies in both the IoMT and IIoT datasets through learning rates, batch sizes, momentum and regularization methods. The following section represents the concept of MBGD and SGD with respect to the integration of the deep learning models.

#### 3.5.1. Mini-Batch Gradient Descent (MBGD)

MBGD is a variation in the traditional gradient descent (GD) that was structured to improve the computational efficiency and learning process of the deep learning models. Instead of customizing the features and parameters of the entire dataset, MBGD uses a subset called a mini-batch for its operation. This allows the deep learning model to update itself more frequently with improved convergence speed and stability. Mathematically, the basic update rule of MBGD can be expressed as

(8) $$\theta_{t+1}=\theta_{t}-\rho \cdot \frac{1}{m}\sum_{i=1}^{m}\nabla_{\theta }L(x^{\left(i\right)},y^{\left(i\right)},\theta_{t})$$

where $\theta_{t}$ is the model parameter at iteration t; ρ is the learning rate; m is the size of the mini-batch, and $\nabla_{\theta }L(x^{\left(i\right)},y^{\left(i\right)},\theta_{t})$ is the gradient of the loss function for the mini-batch sample.

This method combines the advantages of both batch and stochastic gradient descent approaches, allowing the model to converge faster than the traditional GD model. Therefore, this model provides a better and stable learning process compared to SGD.

#### 3.5.2. Stochastic Gradient Descent (SGD)

SGD is also a kind of GD approach where the parameters of the deep learning models are updated after reading each training sample from the dataset. This allows the model to be trained with frequent updates, causing it to move forward from the local minima to attain the best and lowest possible values in the problem. Moreover, these frequent updates can cause fluctuations in the estimations of the loss function and that can result in a slower convergence speed. However, it has the advantage of faster computation per iteration. Therefore, the update rule of SGD can be expressed as

(9) $$\theta_{t+1}=\theta_{t}-\rho \cdot \nabla_{\theta }L(x^{\left(i\right)},y^{\left(i\right)},\theta_{t})$$

SGD is computationally efficient in handling larger datasets, as it updates the weights of the deep learning model in a quick manner. However, these quick updates make the convergence path less smooth, and this can be mitigated by using momentum or adaptive learning rates.

#### 3.5.3. Integration of MBGD and SGD with Deep Learning Models

For CNNs, both MBGD and SGD are used to optimize the weights of the filters that are structured in the convolutional and fully connected layers. Here, the gradient is calculated for a mini-batch in MBGD and single data point in SGD. Therefore, the CNN model can be trained efficiently with spatial hierarchies in the data through efficient parameter optimization. Similarly, LSTMs are designed for sequential data operations, where the gradients of the loss function with respect to weights are calculated for either mini-batches in MBGD or individual time steps in the sequence at SGD. Since LSTMs are operated to capture the long-term dependencies from the dataset, applying this optimization technique would improve the training and convergence rate of the model. In the proposed STID-Net, the weights of the customized convolutional kernels and the fully connected layers are optimized using MBGD or SGD. Similarly, the weights matrices of the LSTM layer on its gates and cell states are also optimized using MBGD or SGD, thereby capturing the temporal dependencies from the sequential data. The mathematical workflow of the utilized MBGD and SGD approaches are descripted in Algorithms 4 and 5.
**Algorithm 4:** Statement for Customized Mini-Batch Gradient Descent (MBGD)**Input:** $\;\;\mathrm{Training}\;\mathrm{dataset}\;X=\{x_{1},x_{2},\ldots ,x_{N}\}$, target labels Y, batch size m$,\;\mathrm{initial}\;\mathrm{learning}\;\mathrm{rate}\;\rho_{0}$, momentum β, regularization factor γ$,\;\mathrm{initial}\;\mathrm{weights}\;W_{0}$. **Output:**   Optimized weights W. $\mathrm{Initialize}\;W_{0}$, momentum term ϑ$=0,\;\mathrm{and}\;\mathrm{learning}\;\mathrm{rate}\;\rho =\rho_{0}$Repeat until convergence:a.Shuffle training data X.b.Partition X$\;\mathrm{into}\;\mathrm{mini}-\mathrm{batches}\;\{X_{1},X_{2},\ldots ,{\;X}_{K}\}$$,\;\mathrm{where}\;\mathrm{each}\;\mathrm{mini}-\mathrm{batch}\;X_{i}$ contains m samples.c.$\mathrm{For}\;\mathrm{each}\;\mathrm{mini}-\mathrm{batch}\;X_{i}$:
i.$\mathrm{Compute}\;\mathrm{the}\;\mathrm{loss}\;L(X_{i},Y_{i},W)$ with regularization:$\;\;\;\;\;L_{reg}\left(X_{i},Y_{i},W\right)=L\left(X_{i},Y_{i},W\right)+\gamma {\left\|W\right\|}^{2}$ii.$\mathrm{Compute}\;\mathrm{the}\;\mathrm{gradient}\;{\nabla_{W}L}_{reg}\left(X_{i}\right)$iii.Update the momentum:$\;\;\;\;\;\vartheta \leftarrow \beta \vartheta +(1-\beta ){\nabla_{W}L}_{reg}\left(X_{i}\right)$iv.Update the weights:       W←W−ρϑv.Adjust the learning rate ρ if a scheduling strategy is applied.
**Algorithm 5:** Statement for Customized Stochastic Gradient Descent (SGD)**Input:** $\;\;\mathrm{Training}\;\mathrm{dataset}\;X=\{x_{1},x_{2},\ldots ,x_{N}\}$, target labels Y$,\;\mathrm{initial}\;\mathrm{learning}\;\mathrm{rate}\;\rho_{0}$, momentum β, regularization factor γ$,\;\mathrm{initial}\;\mathrm{weights}\;W_{0}$. **Output:**   Optimized weights W. $\mathrm{Initialize}\;W_{0}$, momentum term ϑ$=0,\;\mathrm{and}\;\mathrm{learning}\;\mathrm{rate}\;\rho =\rho_{0}$.Repeat until convergence:a.Shuffle training data X.b.$\mathrm{For}\;\mathrm{each}\;\mathrm{sample}\;\left(x_{i},y_{i}\right)\in X:$i.$\mathrm{Compute}\;\mathrm{the}\;\mathrm{loss}\;L(X_{i},Y_{i},W)$ with regularization:$\;\;\;\;\;L_{reg}\left(X_{i},Y_{i},W\right)=L\left(X_{i},Y_{i},W\right)+\gamma {\left\|W\right\|}^{2}$ii.$\mathrm{Compute}\;\mathrm{the}\;\mathrm{gradient}\;{\nabla_{W}L}_{reg}\left(X_{i}\right)$iii.Update the momentum:$\;\;\;\;\;\vartheta \leftarrow \beta \vartheta +(1-\beta ){\nabla_{W}L}_{reg}\left(X_{i}\right)$iv.Update the weights:       W←W−ρϑv.Adjust the learning rate ρ if a scheduling strategy is applied.

Algorithms 2 and 3 indicate the parameters where these optimization algorithms are customized in the proposed work. Here, the learning rate (ρ) is updated dynamically to improve the convergence of the learning models, and it is modified through scheduling strategies. Additionally, the momentum (β) and the regularizations (γ) of both the optimization techniques are customized to provide a stabilized input, accelerating the training process; this leads to the prevention of overfitting by penalizing large weights. Similarly, the batch size (m) is also customized in MBGD for better control over noise and computation balance. The following section explores the effectiveness of the MBGD and SGD optimization techniques in detecting intrusions in the IoMT and IIoT datasets from various deep learning models.

## 4. Experimental Setup

The performance of the proposed work was experimented in Google Colab with the Python program in an Intel Core i7 CPU integrated with an NVIDIA GPU with 32 GB RAM to handle the computational demands of training the deep learning models. A 66:34 train–test split was employed in this work to balance the training robustness of the training models. A k-fold cross-validation method was employed to ensure a consistent performance across folds. Hyperparameters, including the learning rate, batch size, momentum and regularization factors, were fine-tuned by both the MBGD and SGD optimizers and evaluated based on the dynamic and irregular nature of the intrusion patterns in IoT environments. The experimental work was performed with the WUSTL EHMS 2020 [21] and WUSTL IIOT 2021 [22] datasets to estimate the effectiveness of the proposed work on identifying intrusions from both the IoMT and IIoT datasets. In this work, data alteration and spoofing attacks were considered as intrusions of IoMT, and the denial of service (DoS) and reconnaissance were considered as intrusions of IIoT. These particular intrusions are considered in this work as they are the major intrusions of the internet in medical and industrial things. Data alteration is a kind of attack that specifically happens in IoMT where a patient’s data are modified or altered by an attacker which may lead to an incorrect diagnosis or treatment. Similarly, spoofing is also a common attack on IoMT where an attacker impersonates a legitimate device or sensor in the network and provides false data in the system. In general, these attacks can be identified by monitoring for unusual pocket loss, erratic data transfer rates, and irregular packet intervals. Dport, Sport, Loss, Rate and Jitter are the features which are highly efficient in detecting these IoMT attacks.

Similarly, DoS attacks and reconnaissance attacks are the intrusions that are widely present in IIoT, causing network disruptions that lead to permanent network failure or hacking. In a DoS attack, the network devices are overwhelmed with multiple or continuous malicious requests, leading to service disruption. Generally, DoS attacks are identified from an abnormal data transfer rate and improved packet loss. Loss, Rate and Dur are the key features that can work effectively in detecting a DoS attack through neural network approaches. Moreover, reconnaissance is a kind of scanning or probing attack that gathers the behaviour of a network to exploit vulnerabilities at later stages. Dport, Sport and TotPkts are the features that have the ability to detect irregular scan requests from the attackers. Furthermore, in the proposed work, a dimensionality reduction technique is utilized with PCA and RFE to provide the best features in a focused way to the neural network algorithms. Additionally, the neural network algorithms were also experimented with MBGD and SGD to find the best optimizer for the IoMT and IIoT datasets. This optimization allows this work to determine a better learning rate (ρ), thereby ensuring a faster and stable convergence across epochs and avoiding overshooting or underutilizing of the learning process.

### 4.1. Dataset Description

The IoMT dataset originally consists of 16,318 samples, including 14,272 normal and 2046 attack samples. In this work, the original normal samples were kept unchanged, and the attack samples were increased to be around 34% of the overall sample count to create a distribution of 66:34 for normal and attack samples. After applying SMOTE, the attack category is increased to 7087 samples. Table 2 indicates the final data distribution of the IoMT dataset.

In the IIoT dataset, there was originally 972,138 normal samples and 75,033 attack samples. This dataset was also distributed with a 66:34 ratio using the downsampling technique on normal samples and SMOTE on attack samples. After applying SMOTE, the attack category is increased to 356,577 samples, and the overall distribution of IIoT dataset is shown in Table 3.

By utilizing the data alteration techniques like SMOTE and downsampling, this work ensures that the deep learning model is trained with a balanced and robust dataset, thereby ensuring the accuracy of the intrusion detection process. This also reduces the class imbalance and overfitting issues that occurred in the training process of the deep learning algorithms, and it also improves the model generalization, making it highly effective for real-world deployment in intrusion detection applications. Finally, the datasets were split-up, with a ratio of 70:30 for the training and testing process. Table 4 represents the final sample counts considered from each class for the analysis.

### 4.2. Results and Discussion

The performance of the proposed STID-Net was verified with the IoMT and IIoT datasets, where the weights of convolutional, LSTM and fully connected layers are optimized using the MBGD and SGD approaches. In the same way, the performance of the STID-Net was compared with the CNN and LSTM approaches and also with the MBGD and SGD optimizers.

#### 4.2.1. IoMT Dataset Analysis

Table 5 and Table 6 indicate the performance attainments of the verified algorithms on the IoMT dataset with the MBGD and SGD optimizers. The experimental analysis indicates the precision, recall and F1 score of the proposed STID-Net algorithm in comparison to the traditional CNN and LSTM algorithm on all the three classes, including normal, data alteration and spoofing. From Table 5 and Table 6, we observed that the performance of all the algorithms is comparatively better when they are detecting the normal class data over the intrusion data. However, the margin or improvement of the proposed STID-Net is comparatively high when compared to the CNN and LSTM algorithms. We also observed that the performance of the proposed STID-Net is better when it is implemented with the SGD optimizer over the MBGD optimizer.

**Figure 5:**
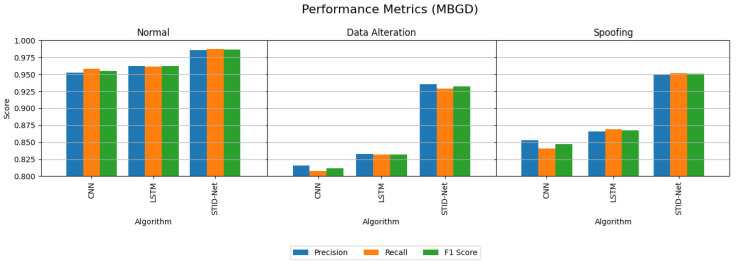
Performance comparison of the verified algorithms in the IoMT dataset with the MBGD optimizer.

**Figure 6:**
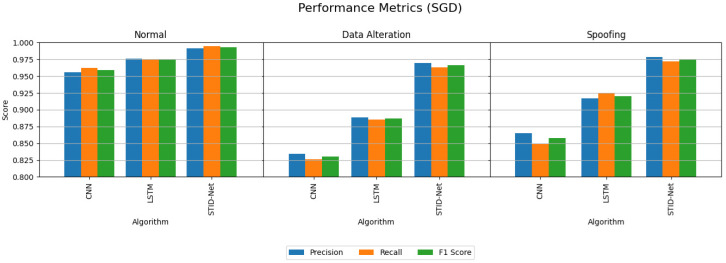
Performance comparison of the verified algorithms in the IoMT dataset with the SGD optimizer.

**Figure 7:**
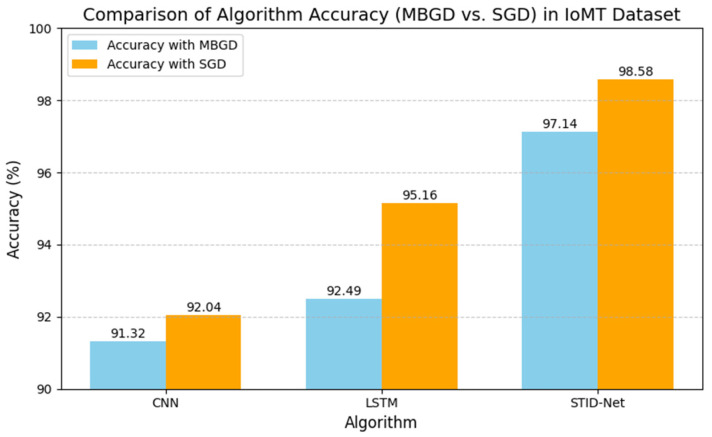
Accuracy of the verified algorithms with the MBGD and SGD optimizers in the IoMT dataset.

The performance deviations between the proposed STID-Net over the other algorithms and the verified optimizers are shown in Figure 5 and Figure 6, where a huge deviation can be observed when detecting intrusion data between all three algorithms. The CNN and LSTM algorithms are sufficient for identifying the normal class data, but there is a problem in those algorithms, specifically in categorizing the intrusion data between the data alteration and spoofing classes. Hence, the overall performance of the CNN and LSTM algorithms is reduced marginally over the proposed STID-Net algorithm. Figure 7 represents the overall accuracy of the verified algorithms on handling the IoMT dataset. The accuracy attainments of the proposed STID-Net are comparatively better than the CNN and LSTM algorithms when using both the MBGD and SGD optimizers. This is achieved as the proposed STID-Net is designed to integrate both spatial and temporal feature sequences from the data. Additionally, the custom kernel size and the attention mechanism improve the overall performance of the proposed concept over the traditional methods.

#### 4.2.2. IIoT Dataset Analysis

The performance attainments of the IIoT dataset with the MBGD and SGD optimizers are projected in Table 7 and Table 8. They show that the performance of CNN and LSTM are comparatively very low when classifying the DOS and Reconn intrusions.

The performance of the CNN and LSTM algorithms over the IoMT dataset on IIoT seems to be low when identifying the intrusions as the number of samples considered in the IIoT dataset is comparatively high compared to the test data. However, this sample size increase does not affect the performance of the proposed STID-Net algorithm. Moreover, the performance of the proposed STID-Net is quite higher in the IIoT dataset when comparing with the IoMT dataset. This is attained by the proposed model as it is trained with a greater number of spatial and temporal features from the training samples.

**Figure 8:**
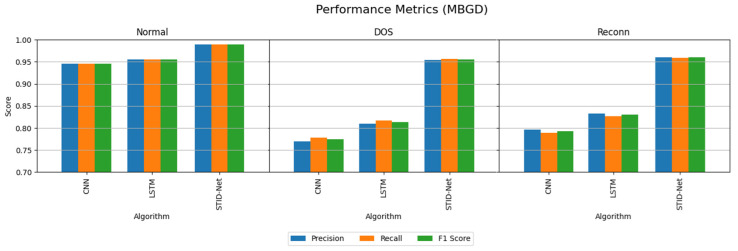
Performance comparison of the verified algorithms in the IIoT dataset with the MBGD optimizer.

**Figure 9:**
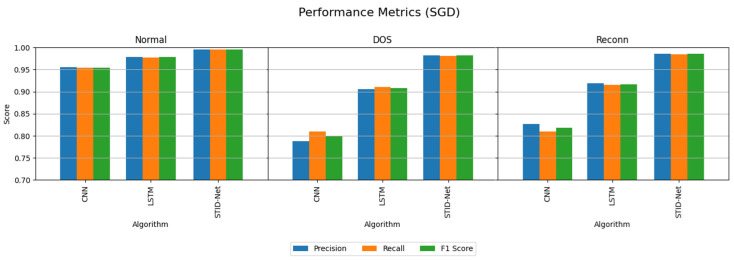
Performance comparison of the verified algorithms in the IIoT dataset with the SGD optimizer.

**Figure 10:**
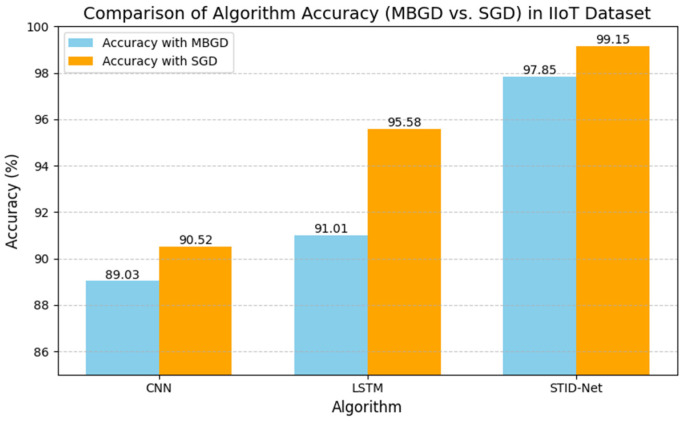
Accuracy of the verified algorithms with the MBGD and SGD optimizers in the IIoT dataset.

The performance deviations on the precision, recall and F1 score of the verified algorithms over the proposed SDIT-Net on the IIoT dataset are shown Figure 8 and Figure 9, where the performance of CNN and LSTM seems to be very low when they are implemented with the MBGD optimizer. However, their performance on categorizing normal data is almost identical, but a noticeable improvement on CNN and LSTM is observed when they are implemented with the SGD optimizer. Though the performance of CNN and LSTM was improved with SGD, it could not meet the performance of the proposed SDIT-Net. SGID-Net stands with good performance metrics in classifying the IoT data in normal and intrusion categories. Moreover, the overall accuracy comparison between the verified models is shown in Figure 10, where the performance of the proposed model with MBGD itself seems to be higher than the CNN and LSTM algorithms with SGD.

#### 4.2.3. Discussion

**Figure 11:**
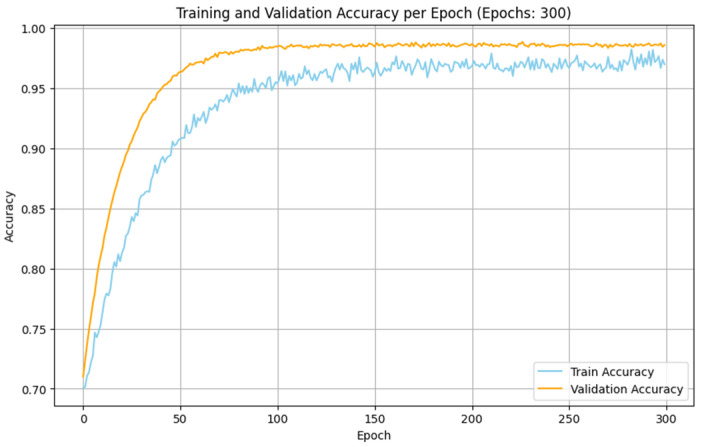
Training and validation accuracy of SDIT-Net with SGD in the IoMT dataset.

**Figure 12:**
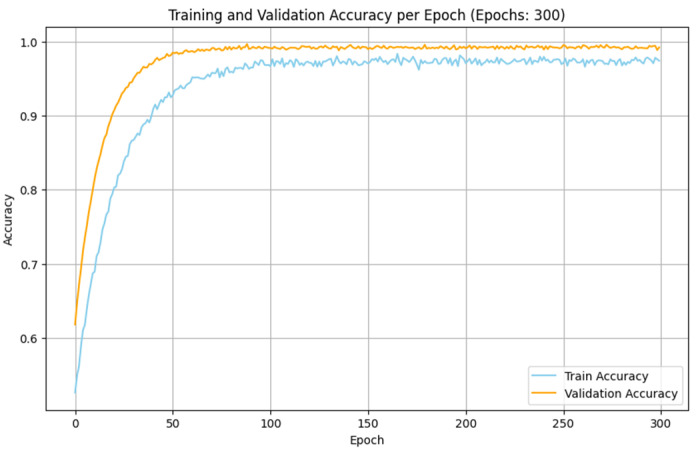
Training and validation accuracy of SDIT-Net with SGD in the IIoT dataset.

The experimental analyses made with the IoMT and IIoT datasets indicate that the performance of the proposed SDIT-Net is satisfied when it is implemented with the SGD optimizer. The training and validation accuracy of the proposed SDIT-Net with the SGD optimizer on both datasets are shown in Figure 11 and Figure 12. This betterment is attained due to the characteristic advantages of SGD on handling a large dataset with complex architectures. Both the IoMT and IIoT datasets are included with many irregular and variable patterns on the intrusion data due to the dynamic nature of the network intrusions and sensor readings. In SGD, the sample-wise weight update on the training process adapts these irregularities in deep learning algorithms by capturing the critical information from the datasets more effectively than MBGD. Moreover, the stochasticity nature of SGD allows the optimization process to estimate the shallow local minima, enabling the exploration of the loss surface in the training process in an efficient manner. This makes SGD more suitable for a hybrid architecture like the proposed STID-Net.

In the proposed STID-Net, the spatial feature extraction is integrated through customized convolutional kernels, and the temporal sequence features are modelled with LSTM layers. Additionally, the attention mechanism provides the model to be an active learner to observe the critical features from the complex input data. Moreover, the attention mechanism assigns dynamic relevance to the extracted features, making them customizable for the convolutional kernels in handling the complex IoT data. Similarly, the finer-grained updates received from SGD are used to enhance the learning process of the STID-Net to ensure precise weight adjustments for the extraction of both spatial and temporal dependencies. Furthermore, the frequent updates received from SGD allow the model to be faster in its convergence, making the model suited for larger datasets like IoMT and IIoT.

The computational performances of the MBGD and SGD optimizers are projected in Table 9. It indicates that the performance of SGD is comparatively better than the MBGD optimizer in terms of both training and inference efficiency. The work also observes an average training time of 108 s per epoch for the IoMT dataset when using the SGD optimizer. It is comparatively 141 s better than the MBGD optimizer. Similarly, SGD achieves a better training of 205 s in the IIoT dataset. Additionally, a better training convergence is also observed with SGD in both datasets, with a difference of 35 and 56 epochs on the IoMT and IIoT datasets, respectively. Similarly, a 61.8% betterment is observed with SGD on its inference time in the IoMT dataset, and a 62.5% inference time improvement is observed in the IIoT dataset. This has been achieved with SGD as it is frequently changing its weights after processing each data sample during the training phase. This frequent weight change mechanism helps SGD to attain a faster convergence rate by making the model avoid shallow local minima from the given samples. Moreover, the stochastic nature of SGD provides a better generalization of the training phase, which helps the model handle complex and high dimensional datasets. The characteristic nature of SGD makes the performance of the STID-Net precise in its weight adjustments, thereby handling spatial and temporal dependencies effectively.

The performance of the proposed STID-Net is compared to the existing methodologies in Table 10 with both the IoMT and IIoT datasets. It indicates that the accuracy attainment of the proposed model outperforms among most of the existing methods. However, a 0.4% improvement is observed with the XGBoost [5] algorithm. Though, the accuracy of XGBoost is higher than the proposed STID-Net, it has been observed that the existing method was evaluated with only 3264 data samples. In order to avoid the biassed outcome, the dataset is augmented in the proposed work and evaluated with 6408 data samples. Additionally, the proposed work highlights its inference time with 107.8 ms; it is comparatives better than the inference time of the extreme gradient boost algorithm at 781 ms. Similarly, a 0.21% improvement is observed in the deep reinforcement Q learning technique [13] on the IIoT dataset, where the total number of features considered in this work was 25 numbers, and in the proposed work, only 15 features were considered. Thus, the proposed work attained an inference time of 149.3 ms.

## 5. Conclusions

The proposed STID-Net effectively addresses the challenges of intrusion detection in both medical and industrial IoT environments with a combination of spatial and temporal feature learning through an attention mechanism. The integration of customized convolutional kernels with LSTM layers made the model more suitable for observing sequential data to identify the irregular patterns of the IoMT and IIoT datasets. The experimental work indicates an accuracy of 98.58% in the IoMT dataset and 99.15% in the IIoT dataset. This indication represents the suitability of the proposed work in real-time security applications in dynamic IoT environments. In the future, the STID-Net would be improved by incorporating it with transformer-based architectures for observing the long-term dependencies in highly complex and large-scale IoT datasets. Additionally, the work will focus on exploring a lightweight and energy efficient model for deploying it in a resource-constrained IoT application. Furthermore, the work will also be focused on the integration of federated learning with a blockchain environment, enabling data privacy along with a better scalability.

## Figures and Tables

**Table 1:** Common features of the IoMT and IIoT datasets.

Features	Description
Dport	The destination port number that indicates the specific service or application on the receiving end.
Sport	The source port number, identifying the specific service or application on the sending side.
Loss	The number of lost packets during data transmissions that can effectively indicate potential network disruptions.
Rate	The data transfer rate, typically in bytes per second, can be used for estimating the speed of communication.
Dur	The duration of the connection or session indicates how long the communication lasts.
SIntPkt	The source interval between packets specifies the time gap between the successive packets sent from the source to the destination.
DIntPkt	The destination interval between packets shows the time gap between packets arriving at the destination from the source.
TotPkts	The total number of packets exchanged during the session specifies the overall traffic volume.
SrcLoad	The load at the source side indicates the amount of traffic or processing load at the source.
DstLoad	The load on the destination side represents the amount of traffic or processing burden at the destination during the transmission.
Load	A measure of network load typically reflects the amount of traffic or demand in the network.
SrcBytes	The total number of bytes sent from the source during the transmission.
DstBytes	The total number of bytes received by the destination during the session.
TotBytes	The total number of bytes transferred in the session, including sent and received data.
SrcJitter	The variation in packet arrival time at the source indicates the irregularity in transmission.
DstJitter	The variation in packet arrival time at the destination indicates network instability, which is more important for detecting intrusions.
pSrcLoss/SrcLoss	The percentage of packet loss at the source mentions the proportion of packets that failed to leave the source successfully or were dropped during transmission.
pDstLoss/DstLoss	The percentage of packet loss at the destination shows the proportion of packets that were not received or were dropped before reaching the destination.
pLoss	The percentage of packet loss indicates the ratio of lost packets to the total number of packets sent.

**Table 2:** Data distribution of the IoMT dataset.

Category	Original Count	SMOTE Count	Final Count	Percentage
Normal	14,272	-	14,272	66.80%
Data Alteration	922	2604	3526	16.50%
Spoofing	1125	2436	3561	16.70%
Total	16,319	5040	21,359	100%

**Table 3:** Data distribution of the IIoT dataset.

Category	Original Count	Downsampled/ SMOTE Count	Final Count	Percentage
Normal	972,138	−27,9544	692,594	66.00%
DOS	68,812	110,065	178,877	17.10%
Reconn	7221	170,479	177,700	16.90%
Total	1,048,171	-	1,049,171	100%

**Table 4:** Data distribution for training and testing process.

Dataset	Class	Training set	Testing Set
IoMT	Normal	9990	4282
	Data Alteration	2468	1058
	Spoofing	2493	1068
IIoT	Normal	484,816	207,778
	DOS	125,214	53,663
	Reconn	124,390	53,310

**Table 5:** Evaluation results of deep learning algorithms with the MBGD optimizers in the IoMT dataset.

Algorithms	Normal			Data Alteration			Spoofing		
	Precision	Recall	F1 Score	Precision	Recall	F1 Score	Precision	Recall	F1 Score
CNN	0.9524	0.9582	0.9553	0.8157	0.8073	0.8115	0.853	0.8412	0.847
LSTM	0.9624	0.9617	0.9621	0.8327	0.8319	0.8323	0.8661	0.8694	0.8677
STID-Net	0.9855	0.9871	0.9863	0.9357	0.9287	0.9322	0.9504	0.9513	0.9508

**Table 6:** Evaluation results of deep learning algorithms with the SGD optimizers in the IoMT dataset.

Algorithms	Normal			Data Alteration			Spoofing		
	Precision	Recall	F1 Score	Precision	Recall	F1 Score	Precision	Recall	F1 Score
CNN	0.9554	0.9621	0.9588	0.8346	0.826	0.8303	0.8652	0.85	0.8575
LSTM	0.9759	0.9748	0.9754	0.8885	0.8851	0.8868	0.9167	0.9245	0.9205
STID-Net	0.9916	0.9948	0.9932	0.9698	0.9634	0.9666	0.9785	0.9721	0.9753

**Table 7:** Evaluation results of deep learning algorithms with the MBGD optimizer in the IIoT dataset.

Algorithms	Normal			DOS			Reconn		
	Precision	Recall	F1 Score	Precision	Recall	F1 Score	Precision	Recall	F1 Score
CNN	0.9456	0.9452	0.9454	0.7698	0.7782	0.774	0.796	0.7888	0.7924
LSTM	0.9557	0.9553	0.9555	0.8097	0.8172	0.8135	0.8332	0.8269	0.83
STID-Net	0.9893	0.9891	0.9892	0.954	0.9567	0.9554	0.9607	0.9587	0.9597

**Table 8:** Evaluation results of deep learning algorithms with the SGD optimizer in the IIoT dataset.

Algorithms	Normal			DOS			Reconn		
	Precision	Recall	F1 Score	Precision	Recall	F1 Score	Precision	Recall	F1 Score
CNN	0.9557	0.9539	0.9548	0.7873	0.8097	0.7984	0.8269	0.8103	0.8185
LSTM	0.9782	0.9778	0.978	0.9053	0.9111	0.9082	0.9192	0.9148	0.917
STID-Net	0.9953	0.9959	0.9956	0.9823	0.9815	0.9819	0.9859	0.9844	0.9852

**Table 9:** Comparative analysis of computational performance for the MBGD and SGD optimizers.

Metric.	IoMT Dataset		IIoT Dataset	
	MBGD	SGD	MBGD	SGD
Average Training Time (per epoch)	249 s	108 s	566 s	361 s
Training Convergence (epochs)	214 epochs	179 epochs	167 epochs	111 epochs
Average Inference Time (per sample)	282 ms	107.8 ms	398.5 ms	149.3 ms

**Table 10:** Comparative analysis of the proposed STID-Net+SGD with existing methodologies.

Dataset	Methodology	Accuracy in %
IoMT	Extreme gradient boosting [5]	95.01
	Gaussian naive bayes [11]	96
	XGBoost [8]	98.98
	Proposed STID-Net	98.58
IIoT	CNN-GR [15]	97.67
	Random forest [9]	99.12
	Deep reinforcement-Q [16]	99.36
	Proposed STID-Net	99.15

## Data Availability

The original data presented in the study are openly available at [16,17].
